# Twitter as a Potential Disaster Risk Reduction Tool. Part III: Evaluating Variables that Promoted Regional Twitter Use for At-risk Populations During the 2013 Hattiesburg F4 Tornado

**DOI:** 10.1371/currents.dis.b305fe1b479528fda724c6f84f546471

**Published:** 2015-06-29

**Authors:** Guy Paul Cooper, Violet Yeager, Frederick M. Burkle, Italo Subbarao

**Affiliations:** College of Osteopathic Medicine, William Carey University, Hattiesburg, Mississippi, USA; College of Osteopathic Medicine, William Carey University, Hattiesburg, Mississippi, USA; Harvard Humanitarian Initiative, Harvard University, Cambridge, Massachusetts; The Woodrow Wilson International Center for Scholars, Washington, DC, USA; College of Osteopathic Medicine, William Carey University, Hattiesburg, Mississippi, USA

**Keywords:** Communications, Disaster analysis, Disaster risk reduction, Prevention and preparedness, Social media, Twitter

## Abstract

Introduction: Study goals attempt to identify the variables most commonly associated with successful tweeted messages and determine which variables have the most influence in promoting exponential dissemination of information (viral spreading of the message) and trending (becoming popular) in the given disaster affected region.

Methods: Part II describes the detailed extraction and triangulation filtration methodological approach to acquiring twitter data for the 2013 Hattiesburg Tornado. The data was then divided into two 48 hour windows before and after the tornado impact with a 2 hour pre-tornado buffer to capture tweets just prior to impact. Criteria-based analysis was completed for Tweets and users. The top 100 pre-Tornado and post-Tornado retweeted users were compared to establish the variability among the top retweeted users during the 4 day span.

Results: Pre-Tornado variables that were correlated to higher retweeted rates include total user tweets (0.324), and total times message retweeted (0.530).  Post-Tornado variables that were correlated to higher retweeted rates include total hashtags in a retweet (0.538) and hashtags #Tornado (0.378) and #Hattiesburg (0.254). Overall hashtags usage significantly increased during the storm. Pre-storm there were 5,763 tweets with a hashtag and post-storm there was 13,598 using hashtags.

Conclusions: Twitter’s unique features allow it to be considered a unique social media tool applicable for emergency managers and public health officials for rapid and accurate two way communication.  Additionally, understanding how variables can be properly manipulated plays a key role in understanding how to use this social media platform for effective, accurate, and rapid mass information communication.

## Introduction

The goal of Part III is (1) to identify the variables most associated with successful tweeted messages and (2) determine which variables have the most influence in promoting exponential dissemination of information (viral spreading of the message) and trending (becoming popular) in the given region. By understanding the variables associated with Twitter messaging, disaster communication managers can purposefully and properly manipulate these factors to promote dissemination to at-risk populations and better understand and react to on the ground changes and events as they occur. This includes identifying those individuals that become ‘superspreaders’ who have a real-time exponential outreach within a local community. Understanding these variables of Twitter offer a way to create and monitor effective communication and information dissemination in real-time during a disaster.

The study utilized a two-fold approach. First, the potential of message virality by variables associated by the top 100 regionally retweeted Twitter users was analyzed and second, the discovery of the overall use of hashtags in the region, a known tactic to stimulate viral Twitter message dissemination.

## Methods

**DATA EXTRACTION AND FILTRATION**

Part II describes the detailed extraction and filtration methodological approach (Figure 1).^1^ The data was then divided into two 48 hour windows before and after the tornado impact with a 2 hour pre-tornado buffer to capture tweets just prior to impact. Tweets and users were analyzed based upon the criteria found in Table 2. The top 100 pre-Tornado and post-Tornado retweeted users were compared to establish the variability among the top retweeted users during the 4 day span (Table 1).

**Filtration Methodology:**
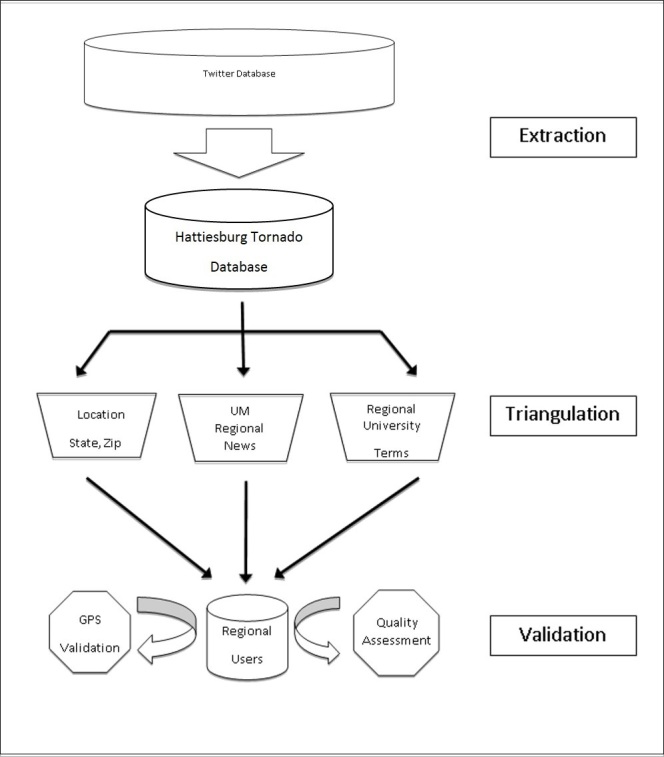
Tweets were extracted and user region was determined through triangulation followed by GPS and quality assessment validation which is described in detail in Part II.

**RETWEETED USER VARIABLES**

Table 2 comprehensively lists the variables that were analyzed and collected through the 48 hours pre- and post- windows. The variables were publicly accessible from any Twitter profile or message. Of note, a Klout score, a commercially accepted (proprietary) rank is used to indicate the influence of a Twitter user as a proprietary variable based mainly on retweets and user-mentions.^2^

**MULTIVARIANT METHODOLOGY**

User data from the top 100 retweeted users was inputted into XLSTAT Version 2013.6.04, a plug-in for Microsoft Excel 2010, and a multivariate analysis was completed by ANOVA using the post-storm retweeted count as the independent variable.^3^^,^ 4 Positive and negative correlations were analyzed requiring p-values <0.05.

The study received an IRB exemption for human subject research from the William Carey University IRB Committee.

## Results

**RETWEETED USER VARIABLES**

Twenty-five of the top 100 post-storm retweeted users are listed in Table 1. Only 9 out of the top 25 (36%) post-Tornado retweeted users were within the top 25 of the pre-Tornado retweets (indicated with a † sign in Table 1). Those 9 retweeted users are the most identifiable superspreaders in the affected region.

**Table 1. Top 25 Users Retweeted by Regional Users:** Top 25 users retweeted by regionally identified Alabama or Mississippi users in pre-Tornado and post-Tornado tweets. † indicates users found in the Top 25 for pre- and post- tornado.

	Pre-Tornado	Post-Tornado
1	@munzly†	@weatherchannel†
2	@wlox†	@nwsjacksonms†
3	@sunherald†	@munzly†
4	@usmgoldeneagles	@hburgamerican†
5	@clarionledger†	@wlox†
6	@jimcantore†	@studentprintz
7	@hburgamerican†	@hattiesburg_ms
8	@nwsjacksonms†	@sunherald†
9	@twitchyteam	@reedtimmertvn
10	@coachphelps	@wunderground
11	@jeffhammondusm	@clarionledger†
12	@16waptnews	@spann†
13	@brentjones4	@jimcantore†
14	@weatherchannel†	@severestudios
15	@iambecauseheis_	@thekegandbarrel
16	@coachtyndallusm	@wdamnickortego
17	@drakee_ymcmb	@msema
18	@severestudios	@ap
19	@spann†	@marshallramsey
20	@southernmisstix	@hpsd
21	@cjusm10	@wcl_shawn
22	@biggoldnation	@caleb_faulkner
23	@info4alerts	@southernmiss
24	@baseballmng	@alastormspotter
25	@usmsoftball	@philbryantms

**MULTIVARIANT RESULTS**

The multivariant analysis (Table 2) revealed several criteria that correlated to higher retweeted user rates. The significant ones, p-values <0.05, are indicated with the † symbol. Variables that demonstrated correlation both pre- and post- Tornado included User follower counts, Retweets that contained a hashtag, Klout Score, retweet included hashtag MSWX, and total user mentions. These variables demonstrate the strongest correlation for possible modification.

Pre-Tornado variables that were correlated to higher retweeted rates include total user tweets (0.324), and total times message retweeted (0.530). Post-Tornado variables that were correlated to higher retweeted rates include total hashtags in a retweet (0.538), and hashtags #Tornado (0.378) and #Hattiesburg (0.254) (Table 2).

**Table 2. ANOVA Correlation of Top 100 Retweeted User Variables:** ANOVA Correlation was calculated using variables from the top 100 retweeted users of regionally identified Alabama and Mississippi Twitter users for pre-48 hours of storm and post-48 hours of storm. † indicates p-values <0.05. ‡ No hashtags were used pre-48 hours

Variable	Pre-48 hours	Post-48 hours
User follower count	0.700†	0.655†
Total user tweets	0.324†	0.155
Total times message retweeted	0.530†	1.000
Retweets that used a hashtag	0.256†	0.630†
Total hashtags in a retweet	0.168	0.538†
User retweeted other users	-0.023	-0.032
Tweets with a hashtag	0.064	0.095
User Klout score	0.398†	0.295†
Used hashtag 'Tornado'	0.134	0.378†
Used hashtag 'Hattiesburg'	-0.037	0.254†
Used hashtag 'MSWX'	0.205†	0.621†
Used hashtag 'Pray for...'	‡	-0.120
Tweeted with multiple hashtags	0.056	0.035
Total user mentions	0.533†	1.000†

**HASHTAG RESULTS**

Overall hashtag usage significantly increased during the storm. Pre-storm there were 5,763 tweets with a hashtag and post-storm there was 13,598 using hashtags. Twenty-five of the top 100 hashtags are listed in Table 3. Values with a † represent p-values <0.05. There was a 0.630 correlation between post-Tornado hashtags and a pre-Tornado hashtags correlation of 0.256 among top retweeted users. Top retweeters that used more hashtags post-Tornado in their retweet also had a correlation of 0.538 with their retweet rate.

**Table 3. Top 25 Hashtags by Regional Users:** Top 25 hashtags used by regionally identified Alabama or Mississippi users in pre-Tornado and post-Tornado tweets. † indicates hashtags found in the Top 25 for pre- and post- tornado.

	Pre-Tornado Hashtag	Total		Post-Tornado Hashtag	Total
1	#smttt†	429		#tornado†	3,170
2	#mswx†	349		#mswx†	2,722
3	#xbox360†	254		#hattiesburg†	2,639
4	#whiteout†	214		#prayforhattiesburg	1,324
5	#beatmemphis†	192		#smttt†	877
6	#ps3†	168		#usm†	656
7	#tornado†	153		#xbox360†	624
8	#usm†	108		#alwx	416
9	#whiteoutmemphis	108		#ps3†	383
10	#mardigras†	95		#southernmiss†	319
11	#wiiu†	93		#mississippi	312
12	#nextgen†	92		#news†	277
13	#reedgreen	87		#whiteout†	214
14	#news†	78		#wiiu†	196
15	#usmbasketball	77		#nextgen†	195
16	#sensitivesaturday	65		#beatmemphis†	192
17	#manga	59		#ms	181
18	#iphone†	56		#hattiesburgtornado	170
19	#nintendo†	55		#wx	166
20	#oomf†	54		#mardigras†	165
21	#mentionsomeoneyo	53		#grammys	143
22	#muchlove	53		#iphone†	140
23	#hattiesburg†	47		#nintendo†	134
24	#thingsyoushouldntd	47		#fb	133
25	#southernmiss†	43		#oomf†	116

## Discussion

Traditional methods of communications have transformed with the development of smart phones and the Internet. Television is slowly being replaced by personal video services such as Netflix, and the radio is being replaced by customizable systems such as Pandora or Spotify.^5^ Disaster notification resources now have to account for the fact that at-risk populations may not be watching local TV or listening to local radio before, during or after a disaster. Expecting these technologically advanced populations to hear sirens or have their local radio or TV on is naïve.^6^^–^ 8 This supports the use of Twitter, as populations remain tethered to their smart phones, and can prove to be more effective at reaching at-risk populations during a disaster.

Twitter provides an instantaneous publically accessible cost effective communication network. The cost is being able to afford a smart phone with access to the Internet—which is now readily available in the United States through state funded programs to socio-economically challenged communities. These research efforts have also demonstrated that Twitter can be purposefully targeted towards the geographically at-risk population if the right messenger is used or if the message is modified appropriately, particularly with the use of hashtags. These findings provide a framework in which Twitter can be utilized in the mitigation and preparedness planning and response phases of the disaster management.

For the disaster plan phase the disaster management team should initially focus on identifying and recruiting the individuals that have large followers in the local region and in building individual disaster response follower bases. User followers along with the Klout score were shown to be the strongest pre-storm significant indicators associated with exponential message distribution. The average Twitter follower has 208 followers but our local superspreaders (out of the top 100 retweeted users) were found to have an average of 1,000 Twitter followers (5x the average follower). Increasing followers for those individuals and agencies involved in disaster response is something that should be expedited as part of the planning process and before large scale events. It should be noted that total number of tweets sent during the event did not correlate with virality so other variables play a stronger role.

In this study it was found that local populations are more likely to follow individuals rather than organizations for crisis news. For example, while the broader local news station WDAM did not generate much activity during the storm, the weather news reporter from WDAM garnered a substantial viral outreach level. It should also be noted that just because one has a popular following does not guarantee viral messaging at the time of the disaster. While these users understand their population’s characteristics, they should be notified of the effective modifiable techniques, particularly the use of the correct hashtag, in order to bolster information relay during a disaster event.

Also observed was an underutilization of individuals who possessed sufficient Twitter communication skills. While these individuals were among the Top 100 retweeted users prior to the storm they did not effectively communicate post-storm. It is speculated that perhaps they were personally and adversely affected by the storm or did not effectively modify their tweets. These individuals need to be targeted for Twitter education and training in the disaster planning phase. Importantly, many were sports reporters who had extensive Twitter experience and a previous history of using hashtags. Religious/spiritual professionals also proved to have large followers but did not necessarily effectively tweet post-storm. Part of the educational process should revolve around encouraging the use of the regional and event driven hashtag. The challenge however is that hashtag usage can evolve rapidly and unexpectedly requiring one to remain nimble with the flow of information.

Retrospectively examining the Hattiesburg and Tornado hashtags showed that the initial hashtag utilized (about 4 hours prior to impact) for the storm was #MSWX (standing for Mississippi), followed by the specific event that occurred #tornado, and later focused on the area it struck (#hattiesburg)(Figure 2). One might conclude that multiple hashtags would be effective for distribution, but this study did not demonstrate that. Purposeful employing of multiple hashtags may prove to bridge evolving hashtag trends and may have statistical significance in twitter message virality in the future.

**Top Hashtag Use:**
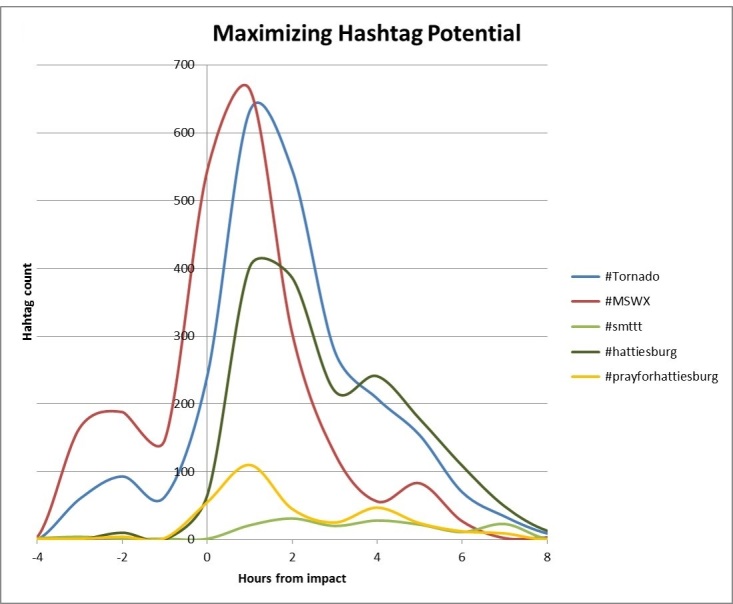
Displays hashtag utilization by regionally identified Alabama and Mississippi Twitter users. 4 hours pre-storm (-4); storm (0); 8 hours post-storm (8);

Lastly, this study suggests that more disaster response applications and opportunities exist for the use of Twitter. Perhaps hashtags could be employed in search and rescue of individuals entrapped after an event such as an earthquake or building collapse. If the individual or their family has access to their phone they could employ a (hashtag) #911 and turn GPS on to allow search and rescue teams to rapidly locate them in real-time. The power of Twitter is an effective bimodal communication in real time with the affected at-risk populations.

This study validates the value of Twitter in disaster planning, mitigation, preparedness and response and possibly in assisting the prioritization of recovery and rehabilitation efforts. It is hypothesized that Twitter can be effectively used in the disaster recovery phase, however our study solely focused on the initial disaster response phase. Future studies should explore Twitter’s potential across the entire disaster cycle.

## Limitations

The findings are limited by the scope of time (96 hrs), and nature of event that was monitored in the region. Regardless the pre-tornado activity was typical of most normal days and activities in the region. The team was still able to extract and triangulate a total of 81,441 tweets and 10,646 twitter users, 27,309 retweets, and 2,637 tweets with GPS coordinates (pre and post).

Search terms only utilized the English language and may miss minor misspellings of those terms not caught in the small user sampling. City names were not used outside of Birmingham and Hattiesburg due to corresponding cities in other states. Social media sites often provide anonymity that many users wish to preserve making defining their region impossible. Some users may be on business accounts and some view tweets without creating accounts.

## Conclusions

Twitter’s unique features allow it to be considered a unique social media tool applicable for emergency managers and public health officials for rapid and accurate two-way communication. Additionally, understanding how and if a variable can be manipulated plays a key role in understanding how to use this social media platform for effective, accurate, and rapid mass information communication. This knowledge will create a better framework for understanding how to create and alter messages that will be effectively received by the at-risk population and potentially mitigate adverse outcomes. This platform can be accessed via a smart phone so is relatively ubiquitous for all populations regardless of social and economic status.

## Competing Interests

The authors have declared that no competing interests exist.
